# Body dissatisfaction, addiction to exercise and risk behaviour for eating disorders among exercise practitioners

**DOI:** 10.1186/s40337-020-00300-9

**Published:** 2020-06-10

**Authors:** Gabriel Lucas Morais Freire, Josy Rawane da Silva Paulo, Adson Alves da Silva, Roseana Pacheco Reis Batista, Juliana Fonseca Nogueira Alves, José Roberto Andrade do Nascimento Junior

**Affiliations:** 1 postgraduate programme in physical education, University Federal do Vale do São Francisco, Petrolina, Brazil; 2 postgraduate programme in psychology, University Federal do Vale do São Francisco, Petrolina, Brazil

**Keywords:** Body dissatisfaction, Eating disorders, Addiction to exercise, Orthorexia, Exercise

## Abstract

**Objective:**

This study investigated the association between body dissatisfaction (BD), addiction to exercise and risk behaviors to eating disorders (EDs) among Brazilian exercise practitioners, besides comparing the variables according to sex, age group and modality.

**Methods:**

Participants were 60 exercice practitioners of fitness (*n* = 44) and crossfit (*n* = 16), with mean age of 26.58 ± 7.76 years. Data collection was conducted through Eating Attitudes Test-26 (EAT-26), Diagnosis of Orthorexia Questionnaire (ORTO-15), Body Shape Questionnaire (BSQ) and Scale of Dedication to Exercise (SDE). Data analysis was conducted through Kolmogorov-Smirnov and independent t tests, Pearson correlation, and Path Analysis (*p* < .05).

**Results:**

Main results showed the association between BD, addiction to exercise and risk behaviour for EDs*.* Further, individuals dissatisfied with their bodies showed higher level of addiction to exercise and risk behavior for EDs. Furthermore, women showed higher presence of BD than men, and fitness participants reported higher presence of addiction to exercise than crossfit practitioners.

**Conclusions:**

This study revealed that BD seems to be a determinant factor for risk behavior for ED’s and addiction to exercise among fitness and crossfit particpants.

## Plain English summary

This study investigated the association between body dissatisfaction (BD), addiction to exercise and risk behaviors to eating disorders (EDs) among Brazilian exercise practitioners, verifying a positive association of BD with addiction to exercise and risk behavior for ED. Further, individuals dissatisfied with their bodies showed higher level of addiction to exercise and risk behavior for EDs, women showed higher presence of BD than men, and fitness participants reported higher presence of addiction to exercise than crossfit practitioners. The authors concluded that BD might be consider a risk factor to determine behavior of EDs and addiction to exercise among fitness and crossfit practitioners.

## Introduction

Body image can be defined as the perception that the individual has in mind about the size, structure, shape and contour of the body, as well as the feelings regarding to these characteristics and the parts that constitute it [1–3]. Fortes, Almeida and Ferreira [4] argue that body image can be focused to thinness, which refers to depreciation of body fat [5], and muscularity-driven, that correspond to the concern with muscle size and volume [1, 4].

Body dissatisfaction (BD) is a disorder of the attitudinal component of body image and includes two spheres: the evaluative, that is characterized by the difference between the current and ideal body image; and affective, that refers to the suffering of the individual due to this difference [6]. BD is a multidimensional construct that can be associated isolated or joint way to weight, body shapes and appearance [1–3]. Alhtough the literature points out that women are more prone to developing BD related to thinness [7–9], some sutides show that men have a higher prevalence of muscle dysmorphia due to the obsessive goal to achieve muscle hypertrophy with minimal body fat [3, 10]. This dissatisfaction can lead to the search for body image transformation through the use of drugs without medical prescription, inadequate nutritional planning, excessive physical exercise and invasive aesthetic procedures [1, 11].

Although it is scientifically proven that the physical exercise provides physical, social and psychological benefits [12, 13], recent studies indicate that excessive practice of physical exercise can take to the development of dependentand pathological behaviours, such as addiction to exercise [14, 15]. Addiction to exercise is characterized by uncontrollable behaviour for the practice of physical exercise, which is manifested by physiological symptoms of tolerance and abstinence and/or psychological symptoms, such as anxiety and depression [16]. This addictive behavior can occur among both athletes and non-athletes [17]. Non-athletes’ addiction to exercise is similar to symptoms of other addictive behaviors, such as changes in mood, loss of behavior control, withdrawal syndrome, excessive time devoted to exercise preparation and recovery, and risk behaviors for eating disorders (EDs) [18, 19].

In recent years, there has been an increase in the number of cases of addiction to exercise among practitioners of different types of physical exercise (e.g. fitnnes and crossfit] [20]. Considering the rise in the number of practitioners of these modalities and the high prevalence of BD in the world population [20, 21], it is relevant to investigate the pathological and dependent behaviors inherent in the search for body aesthetics among fitness and crossfit participants. Fitness training involves strength training and aerobic exercises, and it is considered an effective method to increase muscle strength and mass, improve physical conditioning and lose weight [21, 22]. Another modality of physical conditioning training is crossfit, which combines some functional movements performed at high intensity, including aspects of gymnastics, olympic-style weightlifting and cyclic exercises [20]. Despite the popularity of these types of exercise, few evidences have been found about the psychological characteristics of their practitioners and it is one gap that present study intends to explore [16–18].

BD can also be a suggestive factor for the development of pathological behaviors or addictions, such as risk behavior for EDs [23]. EDs are influenced by a range of factors, such as consumption, disturbed dietary attitudes, and high concern with body aesthetics [15, 24]. These eating patterns might become more extreme and cause adverse consequences for physical, social and psychological health [14, 15]. A frequent EDs among exercise practitioners is orthorexia [25, 26], which corresponds to the excessive commitment to eat healthy [27]. Unlike other EDs, orthorexia is characterized by an excessive concern with healthy food, and not with the amount of food or obsession with the perfect body [28, 29].

The literature supports that BD shows an association with risk behavior for EDs and addiction to exercise [30–32] among practitioners yoga [33], fitness [34] and crossfit [20], indicating that the seek for the perfect body can take the practitioner adopting health risk behaviors [34]. However, this association has never been investigated among Brazilian population practitioners and it is already known that culture, habits, lifestyle and exercise type are factors that interfere directly in this association [26–30].

Thus, this study may provide new relevant information about the association between these variables in a country where millions of people practice exercise every day in order to improve physical appearance. Further, this study advances by investigating such psychological attributes in practitioners of two worldwide popular exercises (fitness and crossfit). Our results may provide relevant information about how BD and perfect body request might associates with the development of addiction to exercise and risk behavior for EDs among two of the most practiced exercises worlwide. Thus, present study aimed to investigate the association between BD, addiction to exercise and risk behaviour for EDs among physical exercise practitioners, in addition to comparing the variables according to sex, age group and modality. Our first hypothesis is that BD will show positive association with both risk behaviour for EDs and addiction to exercise. Our second hypothesis is that women will report to be more dissatisfied with their own body and higher indicative of EDs than men. The third hypothesis is that younger practitioners will demonstrate higher indicative of EDs and BD. The last hipothesis is that fitness practitioners will report higher BD, indicative of EDs and addiction to exercise.

## Methods

### Study design

This is a descriptive study with transversal delineation and methodological research [35]. The study was developed through the guidelines of the Strengthening the Reporting of Observational Studies in Epidemiology (STROBE) [36].

### Setting and participants

The procedures adopted in this research is according to the criteria of ethics in research with human beings according to resolution (466/12) from the National Health Council. Initially, contact was made with managers of the gyms and crossfit boxers in order to obtain authorization for data collection. Then, the Research Ethics Committee of the Federal University of Vale do São Francisco approved the study (protocol 2.442.590). A total of 60 male (*n* = 22) and female (*n* = 38) participants were recruited for the research at gyms and crossfit bexers in the city of Petrolina-PE, Brazil. Participants were selected in a non-probabilistic way and for convenience. The criteria established for the inclusion of the participants were as follow: 1) to be at least 18 years old; 2) to be a physical exercise practitioner for at least 3 months; and 3) to regularly attend the gym/crossfit box at least twice a week. Only the individuals who assined free and informed consent participated of the study. The application of the questionnaires was accomplished individually, in a private room, and participants took approximately 30 min to respond the questionnaires. To avoid sources of bias in the application of the questionnaires, questionnaires were randomized among the participants.

### Measures

#### Demographic information

In order to evaluate the sociodemographic profile of exercise practitioners, a semistructured questionnaire was developed by the authors with questions about modality, age and sex.

#### Eating attitudes Test-26 (EAT-26)

EAT-26 was developed by Garner et al. [37] and it has been frequently used as an outcome measure the frequency of food restriction, binge eating, purging behaviors and environmental pressure for food intake. It is composed of 26 items that are responded on a likert-type scale that vary from 0 to 3 points (always = 3, often = 2, often = 1, rarely = 0, almost never = 0 and never = 0), except for question 4, whose score is reversed (always = 0, often = 0, often = 0, few times = 1, almost never = 2 and never = 3). The total score is calculated from the sum of the responses for each item, ranging from 0 to 78 points. Scores higher than 21 are considered to be indicative of risk behaviour for EDs. It was adapted and validated for Brazilian population by Bigheti et al. [38] the instrument demonstrated acceptable factors of internal and external validity for the Brazilian population. Cronbach’s alpha of the instrument for the present study was α =0.96, indicating strong reliability.

#### Questionnaire for the diagnosis of Orthorexia (ORTO-15)

ORTO-15 has been frequently used as an instrument to measure orthorexia [39]. ORTO-15 proposes to evaluate the frequency of concern with eat too healthy and the level of pathological obsession with correct eating, which can lead to important food restrictions. ORTO-15 consists of 15 items, which are responded on a 4-points likert-type scale with, ranging from always (1) to never (4). According to Domini et al. [39], a total score < 40 is indicative of orthorexic behaviour. It was adapted and validated for Brazilian population by Pontes, Montagner e Montagner [40], providing evidences of the validity and reliability of the instrument among Brazilian population. Cronbach’s alpha of the instrument for the present study was α =0.71, indicating strong reliability.

#### BodyShape questionnaire (BSQ)

BSQ was developed by Cooper et al. [41] and it is a self-completion test with 34 questions which try to evaluate the concern the subject presents with his/her weight and physical appearance. BSQ was adapted and validated for Brazilian population by Di Pietro and Sileira [42]. This questionnaire. The items are responded on a 6-point likert-type scale, ranging from1 (never) to 6 (always). Summing points of each question, classified body dissatisfaction levels according to: less than 80 points = absence of dissatisfaction; from 80 to 110 points = a slight dissatisfaction; from 110 to 140 points = a moderate dissatisfaction; score above 140 points = severe body dissatisfaction. For the present study, BSQ was reorganized into two categories: absence of dissatisfaction - those classified as free from body dissatisfaction; and presence of dissatisfaction - those who were classified as having some level of body dissatisfaction (slight, moderate or severe). After summing the points of each question, the classification of body dissatisfaction levels was carried out according to: less than 80 points = absence of dissatisfaction; from 80 to 110 points = a slight dissatisfaction; from 110 to 140 points = a moderate dissatisfaction; score above 140 points = severe body dissatisfaction. The evaluation was performed by considering the physical fitness and concern expressed during the last 4 weeks of the data collection. Cronbach’s alpha for the instrument was α = 0.95, indicating strong reliability.

#### Scale of dedication to exercise (SDE)

In order to determine the level of addiction to exercise an individual may have with the exercising habit, SDE developed by Davis et al. [36] was applied. The instrument was translated and adapted to Portuguese by by Assunção, Cordás e Araújo [43] and it evaluates the level the wellness sensations are modulated by exercise, the maintenance of the exercise in adverse conditions and the level of interference the physical activity has in social events of the individual. It is a visual analog scale composed of eight questions which range from 0 to 155 mm and therefore, with maximal punctuation of 1.240 mm. The participant has to describe pointing the line that his/her position is and scores higher than 640 indicates high degree of addiction to exercise. Cronbach’s alpha of the instrument for the present study was α =0.70, indicating strong reliability.

### Data analysis

The preliminary analysis was carried out by means of the normality test. Although Kolmogorov-Smirnov revealed a non-normality distribution, skewness and kurtosis indicated a nomal distribution. Thus, we used mean and standard deviation for the characterisation of the results. The independt t test was used for the comparison of BD, addiction to exercise, EDs and orthorexic behavior according to sex (male and female), age range (up 25 years and over 25 years) and modality (fitness and crossfti). The effect size (d) was also calculated using the model proposed by Cohen [44] for differences in the values of two independent groups. According to Cohen’s criteria, a value d = 0.20 represents small effect size; d = 0.50, medium; and d = 0.80, large. The correlation between the variables was performed by Pearson correlation coefficient, and the following values were adopted to interpret the intensity of the correlations: 0.01 to 0.39 = weak; 0.4 to 0.69 = moderate; and 0.7 to 1.0 = strong [45]. The adopted significance level was *p* < .05. Data analysis was conducted through software SPSS version 23.0.

In order to verify the percentage of variance explained of the risk behavior for EDs and addiction to the exercise by BD, a Path Analysis model was conducted through structural equation analysis with the variables that showed significant correlation with BD (*p* < .05). The existence of outliers were evaluated by squared distance of Mahalanobis (DM^2^), which revealed the absence of outliers. Univariate and multivariate normality of the variables were assessed by asymmetry (ISkI< 3.0) and curtosis (IKuI< 10.0). Bollen-Stine Bootstrap technique was used to correct the value of coefficients estimated by the maximum likelihood method [46] implemented in software AMOS 23.0. There were no sufficiently strong correlations between variables that indicated problems with multicollinearity (Variance Inflation Factors< 5.0). According to Kline’s [47] recommendations, the interpretation of the paths was based on the following cutoff: small effect for coefficients <.20, modarate effect for coefficients up to .49 and strong effect for coefficients >.50. In addition, a statistical power analysis in G*Power 3.1.9 [48] for the Path Analysis with one predictor revealed our statistical power to be 83.8% based on our sample of 60 participants, a medium effect size (.15) according to Cohen’s [44] f^2^ criteria, and a .05 *p* value.

## Results

### Participants and descriptive analysis

From the 60 participants of this research, there was a prevalence of women (*n* = 38) and fitness practitioners (*n* = 44). The mean age of the participants was 26.58 ± 7.76 years old. Fitness practitioners (*n* = 44) had mean age of 24.86 ± 7.00 years and crossfit practitioners had the mean age of 31.31 ± 7.98 years. Table 1 demmonstrates that 80.0% of exercise practitioners showed orthorexic behavior, whilst 80.0% had low degree of addiction to exercise, 70.0% had absence of BD and 58.3% showed absence of EDs. The mean scores and standard deviation of each group is also presented in Table 1.

**Table 1:** Presence of BD, addiction to exercise, orthorexic behavior and risk behavior for EDs of the exercise practitioners of the city of Petrolina-PE, Brazil (*n* = 60)

VARIABLE	***f (%)***	M (SD)
**Presence of body dissatisfaction**		
Absence	42 (70.0)	57.16 (12.56)
Presence	18 (30.0)	122.61 (26.01)
**Addiction to Exercise**		
Low level	48 (80.0)	424.62 (135.25)
High level	12 (20.0)	718.54 (36.53)
**Orthorexic Behavior**		
Absence	12 (20.0)	42.91 (2.10)
Presence	48 (80.0)	34.14 (3.91)
**Presence of eating disorder (EAT-26)**		
Absence	35 (58.3)	10.88 (6.30)
Presence	25 (41.7)	61.88 (20.25)

Note: *M* Mean; *SD* Standard-deviation

### Main results

Table 2 presents the comparison of the degree of addiction to exercise, the presence of EDs, the orthorexic behavior and the BD of the exercise practitioners according to sex, age range and modality. It was founf significant difference between men and women only at BD (*p* = .035), indicating that women were more dissatisfied with their own body than men, and the effect size of this difference was large (d = 0.51). There was also found significant difference at the presence of ED’s according to age range (*p* = .016), indicating that younger practitioners (up 25 years old) showed higher indicative of EDs in comparison with practitioners over 25 years old, and the effect size of the difference was medium (d = 0.64). Further, there was significant difference at the presence of EDs according to the type of exercise (*p* < 0.001), indicating that fitness participants showed higher indicative of EDs in comparison with Crossfit practitioners, and the effect size of this difference was large (d = 1.20).

**Table 2:** Comparison of body dissatisfaction, addiction to exercise, presence of EDs and orthorexic behaviour of exercise practitioners of the city of Petrolina-PE, Brazil, according to sex, age and modality

VARIABLES	Body Dissatisfaction	Addiction to Exercise	Eating disorder	Orthorexic Behavior
	M (SD)	M (SD)	M (SD)	M (SD)
**Sex**				
Men	64.36 (28.81)	474.75 (188.07)	36.81 (27.99)	34.27 (5.19)
Women	84.00 (36.42)	488.42 (160.94)	29.42 (29.36)	36.84 (4.79)
	***p*** = .035*	*p* = .767	*p* = .343	*p* = .057
	d = .59	d = .07	d = .25	d = .51
**Age range**				
Up 25 years	77.53 (36.40)	467.06 (176.01)	41.00 (32.46)	36.33 (5.98)
Over 25 years	76.06 (33.97)	499.75 (164.98)	23.26 (21.83)	35.46 (3.973.97)
	*p* = .872	*p* = .461	***p*** = .016*	*p* = .511
	d = 0.04	d = 0.19	d = 0.64	d = 0.17
**Modality**				
Fitness	79.47 (38.07)	482.79 (163.98)	39.31 (30.29)	35.43 (5.42)
Crossfit	68.43 (23.70)	485.09 (191.04)	12.37 (8.68)	37.18 (3.70)
	*p* = .230	*p* = .966	***p*** < .001*	*p* = .164
	d = .34	d = .01	d = 1.20	d = .37

* *Significant difference (p < .05) – Independent t test*

When comparing the indicative of EDs, orthorexic behaviour and addiction to exercise according to BD presence (Table 3), it was found significant difference between groups in the indicative of EDs (*p* = .025) and addiction to exercise (*p* = .048). Thes results indicate that individuals dissatisfied with their own body presented higher presence of EDs and higher score of addiction to exercise, and the effect size of these differences was medium (d = .60 and d = .46, respectively).

**Table 3:** Comparison of orthorexic behavior, presence of eating disorder, and addiction to exercise among exercise practitioners of the city of Petrolina-PE, Brazil, according to BD

Variables	Body dissatisfaction		P	d
	Absence (*n* = 42)	Presence (*n* = 18)		
	M (SD)	M (SD)		
Orthorexic Behavior	36.50 (4.79)	34.50 (5.49)	.190	.19
Presence of eating disorder	26.71 (25.13)	44.77 (33.52)	.025*	.60
Addiction to Exercise	454.33 (163.95)	551.25 (168.62)	.048*	.46

*Significant difference (p < .05) – Independent t test

Table 4 shows the correlation between BD, addiction to exercise, orthorexic behavior and the presence of EDs among exercise practitioners. Orthorexic behavior showed negative, moderate and significant (*p* < .05) correlation with EDs (*r* = −.42). BD showed positive, moderate and significant (*p* < .05) correltation with EDs (*r* = .46), and positive and weak correlation with addiction to exercise (*r* = .23). Such findings indicate that BD has a positive association with the presence of EDs and addiction to exercise.

**Table 4:** Correlation between body dissatisfaction, addiction to exercise, orthorexic behavior and the presence of eating disorder among exercise practitioners of the city of Petrolina-PE, Brazil

Variables	1	2	3	4
1. Orthorexic behavior		−.42*	−.14	−.14
2. Eating disorders			.46*	.05
3. Body dissatisfaction				.23*
4. Addiction to exercise				

*Significant correlation (*p* < .05) – *Pearson Correlation*

**Fig. 1:**
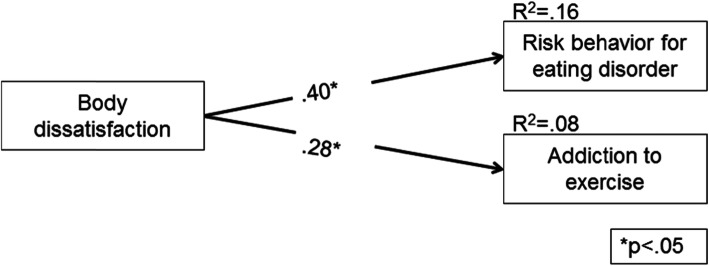
Path Analysis model of the association of body dissatisfaction with risk behaviour for eating disorders and addiction to exercise

In order to verify the percentage of variance explained of the risk behavior for EDs and addiction to exercise by BD, a Path analysis model (Fig. 1) was conducted with variables that showed significant correlation (*p* < .05). BD explained 16% of the variance of risk behavior for EDs and 8% of the variance of addiction to exercise (Fig. 1). It is noted that BD had a moderate and positive effect toward risk behaviour for EDs (β = .40) and addiction to exercise (β = .28). This finding indicates that at each increase of 1 standard deviation at BD unit there is an increase of .40 and .28 standard deviation at risk behaviour units for EDs and addiction to exercise, respectively.

## Discussion

The main findings of this investigation revealed that there is a positive association of BD with risk behaviour for EDs and addiction to exercise (Fig. 1). In addition, practitioners with presence of BD showed higher addiction to exercise and risk behavior for EDs (Table 3). Women were more dissatisfied with their own body than men were, while fitness practitioners and younger individulas (up 25 years old) reported more presence of EDs than crossfit practitioners and older individulas (over 25 years old), respectively (Table 2).

The main outcome is the association of BD with the addiction to exercise and the presence of risk behavior for EDs (Fig. 1), confirming the first hypothesis of this study. This finding reveals that the dissatisfaction with the body can be a potentiating factor for the development of pathological and addictive behaviors, such as addiction to exercise and risk behavior for EDs. These dependent behaviors can be explained by the fact that individuals with less acceptance of their own body are more physically active and, consequently, more susceptible to develop risky behavior for EDs [31, 32]. This finding reveals that BD can be considered a harmful factor for physical and mental health, since it can lead to the adoption of dependent behaviors [49]. Such findings may be related to the fact that aesthetic exercises [e.g. fitness and crossfit] are associated with physical and body development, which can cause higher pressure on the practitioner in pursuit of this goal, and as a consequence, trigger pathological behaviors [30, 50, 51].

Fortes et al. [52] found similar results showing that BD and addiction to exercise are predictor for risk behaviors for EDs among Brazilian athletics athletes. Souza et al. [3] observed the eating behaviour, the physical activity level and the presence of BD among women who practiced resistance training, verifying a prevalence of women with some distortion in body image and a linear association between BD and development of risk behavior for EDs.

It was possible to observe that exercise practitioners dissatisfied with their own body presented higher presence of risk behaviour for EDs and higher degree of addiction to exercise (Table 3). This result also confirms our first hypothesis that that BD can take to the adoption of inadequate eating behavior and the excessive practice of exercise. This finding demonstrates that the ideal of beauty imposed by the media and by society can make people feel more dissatisfied and with the desire to change their bodies [49]. Nowadays, BD has been an important reason for the increase of excessive exercise practice and the adoption of inadequate eating behaviors in both athletes and non-athletes [30, 31, 52]. Palma et al. [6] found similar results investigating BD and addiction to exercise among fitness participants, verifying that participants with high levels of addiction to exercise presented higher body dissatisfaction levels. Clifford e Blyth [53] observed that the duration and frequency of physical exercise during the week are associated with the tendency to develop dependent behaviours among university students.

Currently, BD has been an important reason for the increase of excessive exercise practice and the adoption of inadequate eating behaviors in both athletes and non-athletes [30, 31, 52]. However, our findings demonstrate no significant difference in the presence of orthorexic behavior according to the presence of BD (see Table 3), showing that individuals with absense and presence of BD had high level of orthorexic behavior. It is possible to say that individuals begin the seek for healthy food in order to achieve better health standards and it is not directly associated with the degree of dissatisfaction of the body [54]. Boná et al. [34] found in women with a prevalence of EDs (e.g. anorexia, bulimia, orthorexia) that there was na association between EDs, body image and the practice of physical exercise, however, just like the present investigation, the authors did not find a direct association between the variables. On the other hand, Boná et al. [34] and Ortit [55] observed the association of this type of behavior with body image [weight loss, dissatisfaction with body image]. Considering orthorexia is a new concept in the literature, studies are still scarce and show inconclusive aspects and conclusions, mainly because there is no consensus among researchers about the etiology, diagnostic criteria, symptoms and ways of treating this disorder [15, 26].

Another interesting result is that women reported to be more dissatisfied with their own body than men (Table 2), confirming our second hypothesis. This result can be explained by the fact that women suffer more body changes and hormonal variations along life, which can cause higher BD (34). Further, women are more influenced by sociocultural and psychological factors, such as social medias and judgment of other people [3]. For example, when a woman see a photo of a model in a social media, this situation has a power to take them to the adoption of pathological behavior to achieve that “ideal body” [45]. Costa et al. [31] compared the BD between men and women during gym exercising, verifying similar results to those of this study, with women presenting higher BD presence. Costa et al. [56] conducted a study with Italian exercise practitioners and found that women obtained higher scores of EDs and BD, whilst men presented higher presence of addiction to exercise.

Our data also showed that younger practitioners demonstraded higher indicative of EDs (See Table 2), confirming our third hypothesis. A range of authors argue that age can be an intervening factor for the development of dependent behaviors [57–60]. Adolescence is considered a sensitive period due to increased opportunities that can lead to the adoption of risk behaviors (e.g. sexual behavior, EDs, alcoholic beverages, BD and addiction to exercise) [57, 58]. Bóna et al. [34] observed that age was a predictive factor for the development of EDs among bodybuilding practitioners, especially for younger practitioners. Orrit [55] found that the indicative of muscular dysmorphia was associated with emotional control among young people. Thus, demonstrating that age can be an interveneing factor in the development of EDs and that younger people are more predisposed to these pathological behavors [20, 34]. It is also noteworthy that socio-cultural factors such as culture, habits, lifestyle and type of exercise can also influence the development of dependent behaviors [59, 60].

When comparing the risk behaviour for ED’s, addiction to exercise and BD among fitness and crossfit practitioners (Table 4), it was found that fitness practitioners reported higher indicative of risk behaviour for EDs, confirming partially our last hipothesis. This result can be explained due to the motivation for the practice of physical exercise, given that fitness practitioners normally seek the gym to changes body composition and shape [42]. Crossfit practitioners usually start practices to achieve high performance or attracted by the characteristic dynamics of short group classes [16]. It can be suggested that fitness practitioners already begin the practices at the gym dissatisfied with the body image and, as a consequence, develop risk behaviors for the EDs [6]. Past studies argue that the commitment and frequency of the practice are factors that contribute to the adoption of EDs [30, 61]. Thus, it can be inferred that the difference found in the present study can also be explained by the amount of training of the fitness practitioners, since they practice 3 to 5 times a week, whilst crossfit practitioners take the practices 2 to 3 times a week.

Such findings corroborate those found by Devrim, Bilgic and Hongu [62], who observed some behaviour related to EDs among fitness participants, highlighting that behaviour is directly associated with BD and body dysmorphic disorder. Nevertheless, Lechner et al. [63] observed that college students who practiced aerobic exercises (e.g. crossfit) showed more symptoms of EDs, when compared to practitioners of modalities with more anaerobic characteristics (e.g. weight training and fitness).

Despite the relevant contributions obtained thorugh the results of this study, some limitations need to be raised. First, we highlight the small number of participants and who were practitioners of only two modalities, which makes impossible to generalize the results to practitioners of other exercises, although it brings relevant implications for the professionals involved with the exercise prescription. Thus, future researches should extend the study with practitioners from other regions of Brazilian population and exercises. Another important limitation refers to the cross-sectional design of this research, which does not allow to make inferences of causality between the variables. Perhaps a longitudinal study will be able to point to the causal nature of the association between BD, addction to exercise and risk behavior for EDs. Neverthelees, such limitations are similar to most studies using psychometric instruments among exercise practitioners. In this way, it is also important to understand how the psychometric properties of these measures extend across the exercise context especially in Brazilian population. Further, all instruments are validated to Portuguese language and showed strong internal reliability for the present study. Although the present study has some limitations, this is the fisrt study to investigate the association between these variables in Brazilian practitioners of these specific exercises, which is an important strenght.

## Conclusion

It can be concluded that BD can be considered a determining factor for risk behavior for EDs, as well as for the development of dependent behaviors, such as addiction to exercise, specifically, among fitness and crossfit practitioners. In addition, women showed a higher BD, indicating higher vulnerability to the psychopathological disorders investigated. It was also possible to conclude that younger individuals and fitness practitioners showed a greater tendency to develop risk behaviors for EDs than older individuals and crossfit practitioners, respectively.

From a practical standpoint, the findings suggest some relevant implications for physical education professionals who work with exercise prescription. It is important that instructors and personal trainers analyse the real motives of the individuals to begin the practice of the exercise, always considering the chosen modality. Moreover, it is essential to emphasize the effects of long-term training focusing on maintaining health and quality of life, always respecting the individuality and healthy evolution of training.

## Data Availability

The data supporting the results reported in this article is maintained in the UNIVASF-Universidade Federal do Vale do São Francisco, Department of Physical Education, Petrolina, Pernambuco, Brazil. Please contact author for data requests.

## Abbreviations

BD: Body dissatisfaction
EDs: Eating disorders
